# Total versus inhaled intravenous anesthesia methods for prognosis of patients with lung, breast, or esophageal cancer: A cohort study

**DOI:** 10.3389/fsurg.2023.1155351

**Published:** 2023-04-11

**Authors:** Xiangming Che, Tianzuo Li

**Affiliations:** ^1^ Beijing Obstetrics and Gynecology Hospital, Capital Medical University, Beijing, China; ^2^ Beijing Shijitan Hospital, Capital Medical University, Beijing, China

**Keywords:** breast neoplasia, esophageal neoplasia, lung neoplasia, inhaled anesthesia, intravenous anesthesia, prognosis

## Abstract

**Objective:**

To explore the influences of total intravenous anesthesia (TIVA) and inhaled-intravenous anesthesia on the prognosis of patients with lung, breast, or esophageal cancer.

**Methods:**

In this retrospective cohort study, patients with lung, breast, or esophageal cancer who underwent surgical treatments at Beijing Shijitan Hospital between January 2010 and December 2019 were included. The patients were categorized into the TIVA group and inhaled-intravenous anesthesia group, according to the anesthesia methods used for the patients for surgery of the primary cancer. The primary outcome of this study included overall survival (OS) and recurrence/metastasis.

**Results:**

Totally, 336 patients were included in this study, 119 in the TIVA group and 217 in the inhaled-intravenous anesthesia group. The OS of patients in the TIVA group was higher than in the inhaled-intravenous anesthesia group (*P *= 0.042). There were no significant differences in the recurrence/metastasis-free survival between the two groups (*P *= 0.296). Inhaled-intravenous anesthesia (HR = 1.88, 95%CI: 1.15–3.07, *P *= 0.012), stage III cancer (HR = 5.88, 95%CI: 2.57–13.43, *P *< 0.001), and stage IV cancer (HR = 22.60, 95%CI: 8.97–56.95, *P *< 0.001) were independently associated with recurrence/ metastasis. Comorbidities (HR = 1.75, 95%CI: 1.05–2.92, *P *= 0.033), the use of ephedrine, noradrenaline or phenylephrine during surgery (HR = 2.12, 95%CI: 1.11–4.06, *P *= 0.024), stage II cancer (HR = 3.24, 95%CI: 1.08–9.68, *P *= 0.035), stage III cancer (HR = 7.60, 95%CI: 2.64–21.86, *P *< 0.001), and stage IV cancer (HR = 26.61, 95%CI: 8.57–82.64, *P *< 0.001) were independently associated with OS.

**Conclusion:**

In patients with breast, lung, or esophageal cancer, TIVA is preferable than inhaled-intravenous anesthesia group for longer OS,, but TIVA was not associated with the recurrence/metastasis-free survival of patients.

## Background

Surgery is the backbone and the only potentially curative treatment for solid tumors like breast cancer (1), lung cancer (2), and esophageal cancer (3). Surgery also allows precise tumor staging in terms of tumor size and lymph node involvement (1–3). Surgery necessarily involves anesthesia to render the patients unconscious, blocking pain feeling, and controlling autonomic reflexes (4). Surgical stress causes transient immunosuppression through the release of hormonal mediators such as catecholamines, prostaglandins, and growth factors (5), and prostaglandins and catecholamines can activate receptors involved in the metastatic ability of cancer cells, including B2-adrenergic (6) and cyclooxygenase-2 (7) receptors. In addition, inflammation associated with surgical trauma causes the release of cytokines that can inhibit the natural killer (NK) cells (8), which are essential in the perioperative phase as they are responsible for detecting and destroying circulating tumor cells (9). Furthermore, general anesthesia involves a large number and wide variety of drugs (4), each with possible effects on cancer cells and, subsequently, on patient prognosis (10).

In vitro and *in vivo* studies suggested that volatile inhaled anesthetics could enhance the metastatic ability of cancer cells (11–14), but the exact molecular mechanisms remain incompletely understood. Still, isoflurane, sevoflurane, desflurane, and halothane produce an inflammatory reaction in humans (15). In addition, volatile anesthetics can upregulate the hypoxia-inducible factor 1*α* (HIF1*α*), which confers protective effects on cancer cells (9, 16), and the vascular endothelial growth factor, which can stimulate the growth of cancer cells and neovascularization (11). On the other hand, sevoflurane appears to increase cisplatin sensitivity in lung cancer cells (17). Regarding intravenous agents, propofol is the most commonly used induction and maintenance drug (18). Propofol could have antitumor effects through its anti-inflammatory and antioxidative effects (19–24). Ketamine appears to induce tumor growth by inhibiting NK cells (25, 26). The global evidence about opioids is conflicting among studies and opioids. Some but not all opioids can inhibit NK cells, inhibit the immune system, and accelerate cancer progression (27, 28).

A meta-analysis in 2019 of six studies (>7,800 patients) showed that total intravenous anesthesia (TIVA) was associated with better recurrence-free survival (RFS) compared with volatile anesthetics in breast, esophageal, and lung cancers (29), but there was substantial heterogeneity among the included studies in terms of types of cancer, types of surgery, and patient characteristics. A retrospective study in Northern Europe showed an increased risk of recurrence with volatile anesthetics compared with TIVA in patients with colorectal cancer but without differences in overall survival (OS) (30). Therefore, data is still limited. Therefore, this study aimed to explore the influences of TIVA and inhaled-intravenous anesthesia on the prognosis of patients with lung, breast, or esophageal cancer. The results could hint toward the best anesthesia regimen to improve cancer outcomes.

## Methods

### Study design and patients

In this retrospective cohort study, patients with lung, breast, or esophageal cancer who underwent surgical treatments at Beijing Shijitan Hospital between January 2010 and December 2019 were included. The study was approved by the ethics committee of Beijing Shijitan Hospital. The requirement for informed consent was waived because of the retrospective nature of the study.

The inclusion criteria were (1) proven with lung, breast, or esophageal cancer by postoperative pathological examinations, (2) American Society of Anesthesiologists (ASA) grade I-III, and (3) complete data. The exclusion criteria were (1) underwent preoperative radiotherapy or chemotherapy, (2) underwent emergent surgeries, or 3) underwent secondary surgeries.

The patients were categorized into the TIVA group and inhaled-intravenous anesthesia group, according to the anesthesia methods used for the patients for surgery of the primary cancer.

### Outcomes and data collection

The primary outcome of this study included overall survival (OS), recurrence/ metastasis. OS was the time from treatment to death from any cause. Recurrence referred to the re-appearance of the tumor at the original site. Metastasis referred to the re-appearance of tumors at distant sites.

The baseline characteristics of the patients were collected from the patient charts, including age, sex, body mass index (BMI), tumor stage and type, anesthesia method and drugs, comorbidities, and drug therapy.

### Statistical analysis

SPSS 20.0 (IBM, Armonk, NY, USA) was used for statistical analysis. The Kolmogorov-Smirnov test was used for the normality test of the continuous data. Continuous data with a normal distribution were described as means and standard deviations and compared using the independent t-test. Continuous data not with a normal distribution were described as medians (*P*25, *P*75) and compared using the Mann-Whitney rank-sum test. Qualitative data were compared using the chi-square (*χ*^2^) test. The Cox proportional hazard model was used for multivariable analysis. The Kaplan-Meier survival analysis was used to compare OS and recurrence/metastasis between the two groups, and the log-rank test was used to compare the two groups. All statistical analyses were two-sided, and a two-sided *P *< 0.05 was considered statistically significant.

## Results

### Characteristics of the patients

Finally, 336 patients were included in this study, 119 in the TIVA group and 217 in the inhaled-intravenous anesthesia group. The characteristics of the patients are shown in Table 1. Compared with the inhaled-intravenous group, the TIVA group showed a lower proportion of males (42.0% vs. 58.5%, *P *= 0.004) and a higher frequency of comorbidities (42.0% vs. 26.3%, *P *= 0.003). Moreover, the tumor types of the two groups were statistically different (*P *< 0.001). There were no differences in the other patient characteristics.

**Table 1:** Baseline characteristics of the patients.

Variable, *n* (%)	Total (*n* = 336)	TIVA group (*n* = 119)	Inhaled-intravenous anesthesia group (*n* = 217)	*P*
Sex				0.004
Male	177 (52.7)	50 (42.0)	127 (58.5)	
Female	159 (47.3)	69 (58.0)	90 (41.5)	
Age (years)				0.816
<55	110 (32.7)	38 (31.9)	72 (33.2)	
≥55	226 (67.3)	81 (68.1)	145 (66.8)	
Comorbidities	107 (31.9)	50 (42.0)	57 (26.3)	0.003
Intraoperative use of opioids				0.305
Remifentanil in combination with sufentanil/remifentanil/sufentanil	277 (82.7)	95 (79.8)	182 (84.3)	
Fentanyl in combination with remifentanil/fentanyl in combination with sufentanil/fentanyl	58 (17.3)	24 (20.2)	34 (15.7)	
Postoperative use of opioids	208 (61.9)	72 (60.5)	136 (62.7)	0.695
Muscle relaxant	292 (90.7)	102 (90.3)	190 (90.9)	0.850
Sedative	157 (46.7)	56 (47.1)	101 (46.5)	0.928
NSAID	189 (56.3)	60 (50.4)	129 (59.5)	0.111
Anti-nausea and vomiting drugs	191 (56.9)	63 (52.9)	128 (59.0)	0.285
Intraoperative use of hyperensort				0.234
Methoxamine	305 (90.8)	105 (88.2)	200 (92.2)	
Ephedrine/nradrenaline/phenylephrine	31 (9.2)	14 (11.8)	17 (7.8)	<0.001
**Tumor type**				
Esophageal cancer	112 (33.33)	47 (39.50)	65 (29.95)	
Lung cancer	94 (27.98)	43 (36.13)	51 (23.50)	
Breast cancer	130 (38.69)	29 (24.37)	101 (46.54)	
**Stage**				
I	73 (21.9)	30 (25.4)	43 (19.9)	0.299
II	127 (38.0)	42 (35.6)	85 (39.4)	
III	106 (31.7)	33 (28.0)	73 (33.8)	
IV	28 (8.4)	13 (11.0)	15 (6.9)	

TIVA, total intravenous anesthesia; NSAID, non-steroidal anti-inflammatory drugs.

### Survival

**Figure 1:**
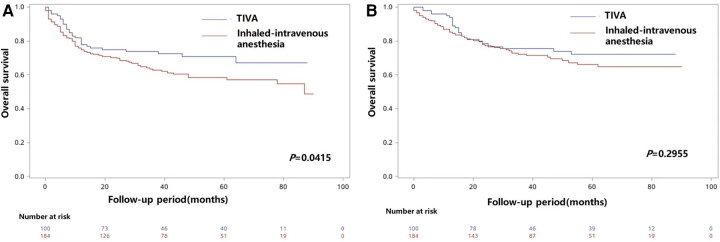
K-M curves of the overall survival (OS) (**A**) and recurrence/metastasis-free survival (**B**) of patients underwent different anesthesia methods.

The OS of patients in the TIVA group was higher than in the inhaled-intravenous anesthesia group (*P *= 0.042) (Figure 1A). There were no significant differences in the recurrence/metastasis-free survival between the two groups (*P *= 0.296) (Figure 1B).

### Factors associated with recurrence/metastasis

The factors influencing recurrence/metastasis are shown in Table 2. Inhaled-intravenous anesthesia (HR = 1.88, 95%CI: 1.15–3.07, *P *= 0.012), stage III cancer (HR = 5.88, 95%CI: 2.57–13.43, *P *< 0.001), and stage IV cancer (HR = 22.60, 95%CI: 8.97–56.95, *P *< 0.001) were independently associated with recurrence/metastasis. The factors influencing OS are shown in Table 3. Comorbidities (HR = 1.75, 95%CI: 1.05–2.92, *P *= 0.033), the use of ephedrine, noradrenaline or phenylephrine during surgery (HR = 2.12, 95%CI: 1.11–4.06, *P *= 0.024), stage II cancer (HR = 3.24, 95%CI: 1.08–9.68, *P *= 0.035), stage III cancer (HR = 7.60, 95%CI: 2.64–21.86, *P *< 0.001), and stage IV cancer (HR = 26.61, 95%CI: 8.57–82.64, *P *< 0.001) were independently associated with OS.

**Table 2:** Influencing factors of recurrence/metastasis of patients.

Variable	Univariable analysis		Multivariable analysis	
	HR (95%CI)	*P*	HR (95%CI)	*P*
**Sex**				
Male	Ref		Ref	
Female	0.33 (0.22–0.51)	<0.001	1.40 (0.79–2.51)	0.253
**Age (years)**				
<55	Ref		Ref	
≥55	1.47 (0.96–2.26)	0.079	0.82 (0.50–1.37)	0.455
**Comorbidity**				
No	Ref		Ref	
Yes	1.32 (0.89–1.97)	0.175	1.53 (0.97–2.41)	0.071
**Anesthesia method**				
TIVA	Ref		Ref	
Inhalational-intravenous anesthesia	1.45 (0.95–2.22)	0.087	1.88 (1.15–3.07)	0.012
**Intraoperative use of opioids**				
Fentanyl in combination with remifentanil/fentanyl in combination with sufentanil/fentanyl	Ref		Ref	
Remifentanil in combination with sufentanil/Remifentanil/sufentanil	1.69 (0.96–2.97)	0.071	0.90 (0.49–1.66)	0.733
**Postoperative use of opioids**				
No	Ref		Ref	
Yes	2.93 (1.85–4.64)	<0.001	1.39 (0.65–2.98)	0.399
**Muscle relaxant**				
No	Ref		Ref	
Yes	5.25 (1.66–16.62)	0.005	2.68 (0.77–9.40)	0.123
**Sedative**				
No	Ref		Ref	
Yes	1.47 (1.00–2.17)	0.049	1.13 (0.74–1.72)	0.564
**NSAID**				
No	Ref		Ref	
Yes	2.21 (1.46–3.33)	<0.001	0.86 (0.50–1.48)	0.590
**Anti-nausea and vomiting drugs**				
No	Ref		Ref	
Yes	2.53 (1.66–3.87)	<0.001	1.05 (0.57–1.94)	0.869
**Intraoperative use of hyperensort**				
Methoxamine	Ref		Ref	
Ephedrine/noradrenaline/phenylephrine	1.74 (0.97–3.12)	0.062	1.60 (0.87–2.97)	0.133
**Stage**				
I	Ref		Ref	
II	2.23 (1.07–4.66)	0.033	3.34 (1.43–7.81)	0.005
III	5.63 (2.74–11.59)	<0.001	5.88 (2.57–13.43)	<0.001
IV	13.70 (6.15–30.51)	<0.001	22.60 (8.97–56.95)	<0.001

HR, hazard ratio; CI, confidence interval; TIVA, total intravenous anesthesia; NSAID, non-steroidal anti-inflammatory drugs.

**Table 3:** Prognostic analysis of the survival of patients.

Variable	Univariable analysis		Multivariable analysis	
	HR (95%CI)	*P*	HR (95%CI)	*P*
**Sex**				
Male	Ref		Ref	
Female	0.32 (0.20–0.52)	<0.001	1.29 (0.67–2.50)	0.447
**Age (years)**				
<55	Ref		Ref	
≥55	1.70 (1.04–2.80)	0.036	1.04 (0.58–1.87)	0.885
**Comorbidity**				
No	Ref			
Yes	1.55 (1.00–2.40)	0.051	1.75 (1.05–2.92)	0.033
**Anesthesia method**				
TIVA	Ref		Ref	
Inhalational-intravenous anesthesia	1.24 (0.78–1.98)	0.360	1.35 (0.79–2.31)	0.280
**Intraoperative use of opioids**				
Fentanyl in combination with remifentanil/fentanyl in combination with sufentanil/fentanyl	Ref		Ref	
Remifentanil in combination with sufentanil/Remifentanil/sufentanil	1.89 (0.97–3.67)	0.062	0.81 (0.40–1.66)	0.570
**Postoperative use of opioids**				
No	Ref		Ref	
Yes	2.36 (1.44–3.88)	0.001	1.05 (0.43–2.53)	0.919
**Muscle relaxant**				
No	Ref		Ref	
Yes	6.11 (1.50–24.94)	0.012	6.16 (0.79–48.09)	0.083
**Sedative**				
No	Ref		Ref	
Yes	1.36 (0.88–2.11)	0.161	1.17 (0.72–1.89)	0.523
**NSAID**				
No	Ref		Ref	
Yes	2.28 (1.43–3.63)	0.001	1.08 (0.57–2.05)	0.811
**Anti-nausea and vomiting drugs**				
No	Ref		Ref	
Yes	1.75 (1.11–2.76)	0.015	0.74 (0.37–1.49)	0.403
**Intraoperative use of hyperensort**				
Methoxamine	Ref		Ref	
Ephedrine/noradrenaline/phenylephrine	2.19 (1.21–3.96)	0.010	2.12 (1.11–4.06)	0.024
**Stage**				
I	Ref		Ref	
II	2.59 (0.98–6.84)	0.055	3.24 (1.08–9.68)	0.035
III	8.71 (3.42–22.18)	<0.001	7.60 (2.64–21.86)	<0.001
IV	17.56 (6.45–47.81)	<0.001	26.61 (8.57–82.64)	<0.001

HR, hazard ratio; CI, confidence interval; TIVA, total intravenous anesthesia; NSAID, non-steroidal anti-inflammatory drugs.

## Discussion

The results suggest that i in patients with breast, lung, or esophageal cancer, TIVA is preferable than inhaled-intravenous anesthesia group for longer OS, but TIVA was not associated the recurrence/metastasis-free survival of patients.In addition, the use of ephedrine, noradrenaline, or phenylephrine during surgery was independently associated with OS.

Surgery and the associated trauma and metabolic stress are well-known to be associated with changes which may causean increased risk of enhancing the metastatic abilities of cancer cells (5–7). The ensuing inflammation will decrease the activity of NK cells (8), leading to a decreased ability to destroy the tumor cells that might be released while manipulating the tumor during resection (9). Hence, intraoperative strategies that could increase the likelihood of the circulating tumor cells being destroyed are clinically relevant.

The tumor has to be resected, and its manipulation is inevitable. Whether cancer cells will detach during resection depends on factors like tumor stage, stage, histological grade, and histological subtype (31, 32). Besides the surgeon's experience in oncological surgery, these factors are mostly non-modifiable. On the other hand, a large number and wide variety of drugs are available for general anesthesia (4), each with possible effects on cancer cells, and it is possible to select the anesthetics to optimize the risk of metastatic spread (10).

Although the exact mechanisms remain mostly unknown, preclinical studies have already demonstrated that inhaled anesthetics enhance the metastatic ability of cancer cells (11–14). This effect could be mediated, at least in part, by inflammation induced by these agents (15), as well as by an upregulation of HIF1*α* that triggers a wide variety of resistance and survival mechanisms in cancer cells (9, 11, 16). Nevertheless, inhaled anesthetics can have other effects on specific cancer. For instance, sevoflurane increases cisplatin sensitivity in lung cancer cells but induces cisplatin resistance in renal cancer (17). The present study was limited to three types of cancer, and the variety of chemotherapeutic drugs used in these patients precluded any subgroup analyses. Nevertheless, all patients received the optimal adjuvant treatments according to the available guidelines (1–3), and it can be assumed that each patient achieved the best outcomes possible.

Regarding intravenous agents, propofol could have antitumor effects through its anti-inflammatory and antioxidative effects (19–24), but ketamine appears to induce tumor growth by inhibiting NK cells (25, 26). Opioids seem to have various effects on cancer cells that depend upon the type of opioids in specific cancer types (27, 28). In the present study, remifentanil was not associated with OS or recurrence/metastasis compared with fentanyl.

The present study showed that TIVA was associated with a better prognosis than inhaled-intravenous anesthesia in patients with breast, lung, or esophageal cancer. It is supported by a recent meta-analysis of six studies involving >7,800 patients with breast, esophageal, and lung cancers, with better RFS with TIVA than with inhaled anesthesia (29). Of note, the previous meta-analysis involved substantial heterogeneity in its analyses, and the results must be taken cautiously and confirmed. A retrospective study in Northern Europe showed an increased risk of recurrence with volatile anesthetics compared with TIVA in patients with colorectal cancer but without differences in overall survival (OS) (30). In this study, similarly, we found that inhaled-intravenous anesthesia was independently associated with recurrence/metastasis, which was consistent with the retrospective study in Northern Europe. We believe the reason why conclusion of this present study was different from previous researches could possibly be that the patients were from a single center and the resulting sample size was small, which needs to be improved in future study. Large studies on breast, gastric, liver, colon, and rectal cancers have indicated increased mortality in cancer patients undergoing inhalation anesthesia (33–37). The lack of association between anesthesia type and OS could be attributed by postoperative complications like wound complications, pulmonary infections, and anastomotic leaks (38, 39), as well as short and long-term complications associated with adjuvant treatments (40, 41). The results could also be influenced by relativelyshort follow-ups, preventing the observation of long-term events. In addition, older patients with comorbidities have a risk of dying from causes other than cancer (42, 43). Retrospective studies of patients with esophageal cancer showed increased mortality with inhaled anesthesia but fewer myocardial infarctions, which could influence OS (37, 44, 45). Hence, the lack of association between inhaled anesthesia and OS could be due to a decrease in other postoperative events such as myocardial infarctions. It was worth noting that in the past before BIS monitoring was popular, more short-term surgeries were done using TIVA. BIS had been used to monitor the depth of anesthesia since 2010 in this study center; unfortunately, relevant data were not collected during this study. However, we found that there was no significant difference in tumor stage between the two groups, which means that the complexity of surgery was equal for both groups.

The use of ephedrine, noradrenaline, or phenylephrine during surgery was associated with increased mortality in patients with gastric cancer (46). In the present study, the use of ephedrine, noradrenaline, or phenylephrine during surgery was independently associated with OS. This association is probably associated with greater hemodynamic instability in the patients, but this parameter was difficult to assess from the patient charts.

Previous data analysis has found that differences in anesthesia type do exist, such differences as recurrence and metastasis outcomes and OS among different tumor type distribution. Therefore, variable of tumor type was included in the multivariate regression in the prognostic analysis. And the results showed that after tumor type and other factors were adjusted, the risk of recurrence and metastasis was increased in the intravenous-inhalational anesthesia group compared with the intravenous anesthesia group (OR = 1.88, 95%CI: 1.15–3.07).

In the present study, the tumor stage was the strongest factor associated with recurrence/metastasis and OS. These results go with common sense since the tumor stage is an important prognostic factor for patients with cancer and is among the most widely used prognosis system (1–3). Nevertheless, the results suggest that inhaled-intravenous anesthesia is a risk factor for OS that is independent of the tumor stage.

This study has limitations. It was a retrospective study limited to the data available in the patient charts, and it has the inherent selection bias of retrospective study. The patients were from a single center, and the resulting sample size was small. Additional prospective, multicenter, randomized controlled trials with larger sample sizes are needed to provide high-grade evidence.

In conclusion, the results suggest that in patients with breast, lung, or esophageal cancer, TIVA is associated with relatively higher OS than inhaled-intravenous anesthesia, while we did not find the association between TIVA and the recurrence/metastasis-free survival of patients. The use of ephedrine, noradrenaline, or phenylephrine during surgery was independently associated with OS.

## Data Availability

The original contributions presented in the study are included in the article/Supplementary Material, further inquiries can be directed to the corresponding author/s.
